# Kopernio

**DOI:** 10.5195/jmla.2019.805

**Published:** 2019-10-01

**Authors:** Matthew B. Hoy

**Affiliations:** Associate Director, Mayo Clinic Libraries, Mayo Clinic, Rochester, MN, hoy.matt@mayo.edu

## INTRODUCTION

A major challenge medical librarians face is connecting their patrons to full-text journal articles. Tools like link-resolvers and full-text databases can provide access to thousands of journals, but for these tools to work, patrons must begin their searches via their libraries’ websites. If library patrons search the open web and find a journal article on a publisher website, they do not have clear path to full-text access. Kopernio is a browser plugin aimed at solving this problem. This review describes what Kopernio is and how to set it up and explores some of its advantages and disadvantages. A brief discussion of similar products is also included.

## WHAT IS KOPERNIO?

Kopernio is a browser extension that simplifies finding full-text portable document format (PDF) copies of journal articles. The main interface is an icon overlaid on pages containing journal article information. If the web page a patron is using contains a journal article digital object identifier (DOI), Kopernio will attempt to locate a link to a PDF copy of that article. It does this by querying a list of known sources for articles, including publisher websites, open access repositories, and Google Scholar. If it can access a copy of the PDF, it will notify the user by changing the icon in the lower left corner of the screen. For some databases, including PubMed, Kopernio also integrates download icons into results lists, giving a quick and clear indicator which articles in a particular set can be easily accessed.

All users are also given a “locker,” 1,000 megabyte (MB) of online storage space associated with an account that users can create. Any articles retrieved via Kopernio are automatically stored in this locker for future reference. Articles stored in the locker can also be tagged and cited using a built-in citation tool. Users can also select a preferred search tool to integrate with the Kopernio toolbar button. Google, Web of Science, and PubMed can all be searched directly from the Kopernio button on the browser toolbar. Other available settings include preferred citation manager and citation styles.

One important feature of Kopernio is that users are always presented with the most authoritative version available of an article. Official publisher PDFs are given precedence over repository copies, and all PDFs are sourced from legitimate sources.

## SETUP

Kopernio requires minimal configuration. Once users have down loaded the extension in their browsers of choice, they will be asked to provide an email address and choose their home institutions. If their institutions have registered proxy information with Kopernio, user requests will be routed through that proxy. Users can log in to their institutional proxy once, and Kopernio will encrypt and store credentials in the browser, which speeds up the article retrieval process. Users can also select a preferred search tool to integrate with the Kopernio toolbar button.

## SIMILAR PRODUCTS

There are several other tools that serve a similar purpose as Kopernio:

- Nomad is a browser extension built by Third Iron, the makers of Browzine. Nomad functions similarly to Kopernio, adding icons to pages that contain journal article information indicating whether or not access is available. One clear advantage that Nomad has over Kopernio is that it is aware of the user’s affiliation and has access to library holdings data; however, Nomad is not free. Libraries must purchase a yearly license to offer the tool to their users.
- Unpaywall is a tool developed by Impactstory that focuses on finding open access journal articles. Similar to Kopernio, Unpaywall overlays an icon on the user’s screen that indicates when a free, open access copy of an article is available. Unpaywall was reviewed in the April 2019 issue of the *Journal of the Medical Library Association*.
- Google Scholar Button and the OA Button insert a button onto the user’s toolbar. When the user finds a web page containing a journal article DOI, they can click the toolbar button to locate a copy. Both tools search a number of sources for free and open access copies of journals articles.

## CONCLUSION

Kopernio is an extremely useful tool for users who search for journal article PDFs. The most appealing functions are the clear visual indicators for downloads and the ability to proxy links for users even if they do not find journals via the library website. The locker, citation management, and tagging features are a nice bonus but will probably only be of interest to power users.

Librarians will want to be sure they provide information about their institutions’ proxies to Kopernio, so that their users can benefit from this tool. Additional information about Kopernio and instructions for librarians to provide their proxy information can be found at Kopernio for libraries.

## 

***Matthew B. Hoy, AHIP****, hoy.matt@mayo.edu, Associate Director, Mayo Clinic Libraries, Mayo Clinic, Rochester, MN*

